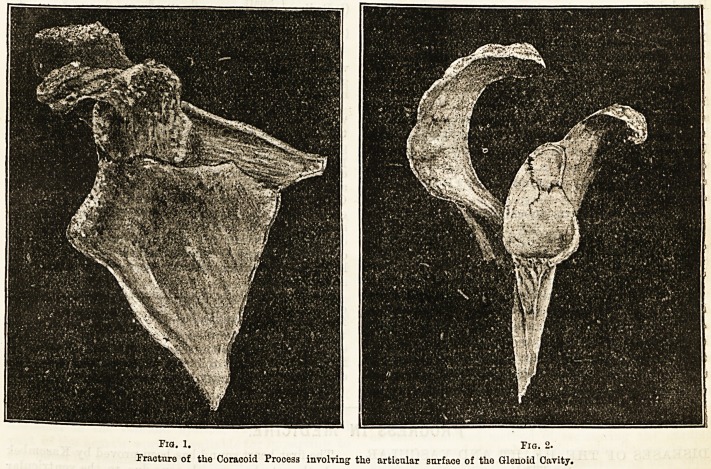# The Treatment of Fractures by Some of the Simpler Methods

**Published:** 1894-08-25

**Authors:** S. Edwards Jones

**Affiliations:** late Senior Resident Surgeon Glasgow Royal Infirmary


### Medical Progress and Hospital Clinics.

### THE TREATMENT OF FRACTURES BY SOME OF THE SIMPLER METHODS.—I.

By S. Edwards Jones, L.R.C.S.Ed., L.R.C.P.Ed., &c., &c., late Senior Resident Surgeon Glasgow Royal Infirmary.

Notes on 105 Cases, with Results.

The greater number of cases which I report came under my notice during the last twelve months, whilst a resident surgeon at the Glasgow Royal Infirmary. The treatment which I applied is, with some exceptions, the one advocated by my old teacher, Professor Knox, and, from the simplicity of application and its efficiency, combined with economy, it is worthy of the consideration of all surgeons in general practice. It will be seen that, with the exception of long Liston splints and rectangular ones (the latter of which might also be dispensed with), the majority of the fractures were treated by means of poroplastic and gooch, materials of a portable nature, which can be fitted to any individual case; whilst the expensive and elaborate apparatus recommended in text books are often, when most required in country practice, unattainable. The results appended will also, I think, compare very favourably with the statistics at our disposal.

**Fractures of the Clavicle (9 cases).**—In seven of the cases the fracture was caused by indirect violence; in the other two, a man æt. 35 and a woman æt. 26, the cause was direct.

*Situation of Fracture.*—In eight of the cases the fracture was at or near the junction of the two curves; in the other case, between the conoid and trapezoid, and caused directly.

*Treatment.*—With the exception of one case—a fracture of both clavicles, in which the man was kept for three weeks in the recumbent position—treatment was by means of a soft axillary pad fastened over the opposite shoulder; a bandage simulating the application of plaster in Sayre's method, but with not so much elevation of the point of the elbow, was then applied.

*Results.*—In one or two very oblique fractures there was some resulting deformity, but in the remainder the amount of callus thrown out was small, and in the case of the double fracture the result was also very satisfactory.

Fig. 1.

Fig. 2.

Fracture of the Coracoid Process involving the articular surface of the Glenoid Cavity.

**Fractures of the Scapula (2 Cases).**—One of these cases, a fracture of the coracoid process, came under my notice three years ago, in private, and I now take this opportunity of reporting it, as at that time it was diagnosed as having also an involvement of the neck of the scapula. The other case was verified by post-mortem examination. I append, on account of their rarity, a short clinical report.

I. *Fracture of the Neck of Glenoid Cavity and Coracoid Process.*—Mrs. K., æt. 64. April 20th, 1891. Whilst carrying a bundle on her head, and being intoxicated, she fell, coming violently to the ground on the point of her left shoulder. When I saw her some hours later she complained of great pain and inability to raise the arm. On placing the hand over shoulder and moving arm crepitation easily made out.

Head of bone displaced, simulating a dislocation, but attempts at reduction caused great pain and grating sensation, and although head of bone seemed to get into position, it returned immediately. Being a thin, spare woman, the coracoid process could be felt to move on any interference with the arm. There was distinct lengthening.

*Treatment.*—Reduction by extension, pad in axilla, and bandaging of arm to the side.

*Result.*—Not very good, as there was some atrophy of deltoid and a great deal of stiffness, requiring a long course of massage and manipulative interference. Seen two years afterwards there was still some atrophic change, but a fairly useful joint.

II. *Fracture of the Coracoid Process involving the articular surface of the Glenoid Cavity.* (See figs. 1 and 2).—Mrs. C. H., æt 52, June 17th, 1893. Admitted to the Royal Infirmary, having been picked up in an unconscious condition at the bottom of a stair. She died from head injuries five days after admission. On examination she presented the usual signs of fracture of this process.

Post-mortem examination revealed three distinct fractures of the base, a dissection of the shoulder showing the condition as illustrated. Head of humerus was intact. I must thank my friend, Mr. D. Lauder Lindsay for these drawings from the original specimen, which is now in Professor Knox's private museum. The illustrations only show a portion of the scapula.

**Fractures of the Humerus (14 Cases).**

| | No. | Sex. | | Age. | |
|---|---|---|---|---|---|
| | | Male. | Female. | Under 40. | Over 40. |
| Surgical Neck | 1 | 0 | 1 | 0 | 1 |
| Shaft | 10 | 8 | 2 | 4 | 6 |
| Lower End | 3 | 2 | 1 | 3 | 0 |

*Treatment.*—(*a*) Surgical Neck: A wedge-shaped axillary pad; a poroplastic shoulder cap, and with the arm bandaged to the body, was the treatment applied in this case. (*b*) Shaft: A well padded internal rectangular splint; two smaller splints of gooch, and if the fracture were high the addition of a shoulder cap were the means employed. In all cases bandaging from the hand, care also being taken that the sling employed should not cause tilting of the elbow; in fact only the distal part of the forearm should be included in the sling. (*c*) Lower End: Two well padded rectangular poroplastic splints moulded, and extending from the fingers to the middle part of the arm. This apparatus was taken down in three weeks, and passive movement commenced. The three fractures of this variety occurred in young children, and were accompanied by a great deal of swelling, necessitating the use of Lotio plumbi before being set.

*Results.*—In two very oblique fractures of the shaft the apparatus was left on for five weeks, followed by the use of starch bandages. The results in all the cases were good and all that could be wished for.

**Fractures of the Forearm (25 cases).**

| | | No. | Sex. | | Age. | |
|---|---|---|---|---|---|---|
| | | | Male. | Female. | Under 40. | Over 40. |
| (*a*) Radius and Ulna | | 13 | 11 | 2 | 10 | 3 |
| (*b*) Radius | Shaft | 5 | 2 | 3 | 2 | 3 |
| | Colles'. | 4 | 3 | 1 | 3 | 1 |
| (*c*) Ulna | Shaft | 1 | 1 | 0 | 1 | 0 |
| | Olecranon | 2 | 0 | 2 | 2 | 0 |

In twenty of the cases the fracture was due to direct violence, two of the cases being compound and two greenstick; in the five remaining cases being caused indirectly, one of the patients being nearly 80.

*Treatment.*—In all cases of fracture of the forearm, with the exception of that of the olecranon process, the treatment was by means of anterior and posterior gooch splints, wooden parts being internal. The posterior splint was the longer one, extending to the ends of the fingers, whilst a part was cut out in the anterior splint for the ball of the thumb. In cases of Colles' fracture both splints were shortened at the end of nine days, the anterior reaching into the palm so as to allow of movement of the fingers. The two cases of fracture of the olecranon were treated by an open angle splint placed anteriorly, the forearm lying in a position midway between pronation and supination.

*Results.*—In all the cases the results were very good excepting a sailor with a Colles', who did not return for a month after the fracture was set, with the result that there was a prolonged after treatment. I was especially pleased with this method of treating Colles' fracture on account of its simplicity and the difficulties experienced by so many surgeons.

Wrexham.

## Figures and Tables

**Fig. 1. Fig. 2. f1:**